# Population structure, relatedness and ploidy levels in an apple gene bank revealed through genotyping-by-sequencing

**DOI:** 10.1371/journal.pone.0201889

**Published:** 2018-08-15

**Authors:** Bjarne Larsen, Kyle Gardner, Carsten Pedersen, Marian Ørgaard, Zoë Migicovsky, Sean Myles, Torben Bo Toldam-Andersen

**Affiliations:** 1 Department of Plant and Environmental Sciences, University of Copenhagen, Frederiksberg C, Denmark; 2 Department of Plant, Food and Environmental Sciences, Dalhousie University, Faculty of Agriculture, Agricultural Campus, Truro, NS, Canada; University of Guelph, CANADA

## Abstract

In recent years, new genome-wide marker systems have provided highly informative alternatives to low density marker systems for evaluating plant populations. To date, most apple germplasm collections have been genotyped using low-density markers such as simple sequence repeats (SSRs), whereas only a few have been explored using high-density genome-wide marker information. We explored the genetic diversity of the Pometum gene bank collection (University of Copenhagen, Denmark) of 349 apple accessions using over 15,000 genome-wide single nucleotide polymorphisms (SNPs) and 15 SSR markers, in order to compare the strength of the two approaches for describing population structure. We found that 119 accessions shared a putative clonal relationship with at least one other accession in the collection, resulting in the identification of 272 (78%) unique accessions. Of these unique accessions, over half (52%) share a first-degree relationship with at least one other accession. There is therefore a high degree of clonal and family relatedness in the Danish apple gene bank. We find significant genetic differentiation between *Malus domestica* and its supposed primary wild ancestor, *M*. *sieversii*, as well as between accessions of Danish origin and all others. Using the GBS approach allowed us to estimate ploidy levels, which were in accordance with flow cytometry results. Overall, we found strong concordance between analyses based on the genome-wide SNPs and the 15 SSR loci. However, we argue that GBS is superior to traditional SSR approaches because it allows detection of a much more detailed population structure and can be further exploited in genome-wide association studies (GWAS). Finally, we compare GBS with SSR for the purpose of identifying clones and pedigree relations in a diverse apple gene bank and discuss the advantages and constraints of the two approaches.

## Introduction

Apple germplasm diversity has been explored and described for decades using low-density markers. Here, simple sequence repeat (SSR) markers have been the preferred approach to characterize and compare the germplasm kept in several European apple collections [1]. The heritable, co-dominant information of SSR markers makes them powerful tools for exploring apple gene bank collections in order to reveal genetic diversity, pedigrees, and mislabelled accessions. However, recently introduced genome-wide marker systems provide alternatives to low density marker systems for genotyping.

High-density marker systems based on single nucleotide polymorphisms (SNPs) are useful because they allow genome-wide comparisons which can reveal small genetic differences between individuals that are otherwise quite similar. In apple, medium density Illumina Infinium arrays containing 8k and 20k SNPs were initially developed [2, 3], followed recently by a high-density 487k SNP Affymetrix Aciom array [4]. SNP arrays allow for the investigation of genetic variation, genome-wide association studies (GWAS), genomic selection [4] and deducing the mosaic founder composition of cultivars through reconstruction of pedigrees [5]. However, they are relatively expensive to use and may result in poor hybridization in diverse perennial crops [6]. The development of new next-generation sequencing techniques, such as genotyping-by-sequencing (GBS) protocols [7], allow the simultaneous discovery and genotyping of markers. In comparison to the development of SNP arrays, GBS offers a reduced cost by enabling marker discovery and genotyping in a single step in high diversity species like apple. Moreover, GBS is even applicable in species for which no reference genome is available. GBS was recently applied to characterize a large apple collection in the USA [8].

Important breeding material is often kept in gene bank collections where a lack of genomic information and scarce documentation of agronomic traits pose serious threats to the potential utilization of germplasm resources. Phenotyping of diverse gene bank material is essential for identifying accessions with superior traits for breeding purposes. Phenotype data collected from gene banks can be paired with genome-wide marker information to facilitate genomics-assisted breeding [8, 9]. Genomics-assisted breeding is especially valuable in tree crops with long juvenile phases, such as apple, where genetic screening at the seedling stage may replace several years of the traditional breeding process [10–12]. Genotyping is also a useful tool for verifying the identity of accessions, especially in old gene bank collections that have been renewed and replanted several times, increasing the risk of curation error and thus misidentification. Finally, genotyping is a valuable tool for identifying clones, since frequent incorrect identification, clonal selections, inaccurate passport information and lack of historical documentation complicates apple classification [13, 14].

Here, we used GBS to genotype a collection of 363 apple accessions, including 14 *Malus sieversii* accessions, belonging to the Pometum gene bank collection (University of Copenhagen, Denmark). This is the most comprehensive collection of local Danish apple cultivars, which we recently studied using SSR markers and flow cytometry [15]. We use GBS to generate further insights into the population structure, relatedness and ploidy levels as well as compare the strengths of this high-density, genome-wide marker information with low density SSR markers.

## Materials and methods

### Plant material and SNP genotyping

We sampled 349 *Malus domestica* and 14 *M*. *sieversii* accessions (S1 Table) belonging to the Pometum (University of Copenhagen, Denmark). Young leaves from vigorously growing shoots were sampled. One leaf from each accession was immediately transferred to silica-gel and stored in individual airtight plastic bags. Extraction, quantification and further procedures were performed in 96-well plates. DNA extraction was performed using the DNeasy® 96 Plant Kit (Qiagen®, Hilden, Germany) following the manufacturer’s protocol. Total DNA content was quantified with the dsDNA dye (Promega) on the Agilent Mx3005P QPCR System. GBS based on Elshire et al., 2011 [7] using the enzyme *Ape*K1 and 96 samples multiplexed was performed by the Biotechnology Resource Centre at Cornell University, USA using 100 bp long single-end reads on HiSeq2000 (Illumina, San Diego, CA).

Raw sequence data was first parsed with a custom python program (see [16]) to split the single multiplexed fastq file into 96 separate fastq files indexed by GBS barcode. During the splitting process several quality control procedures were implemented including (1) discarding sequences with ambiguous bases in the barcode or restriction remnant, (2) 3' adapter trimming (i.e. if the genomic fragment was less than ~100 base pairs in length), (3) detection and trimming of chimeric sequence (by examining reads for a second restriction site and discarding any reads where a restriction site was present), and (4) discarding any trimmed sequences less than 30 bp in length. Individual fastq files were then independently aligned to the Malus 1.0p reference genome (www.rosaceae.org) with bwa 0.6.1 [17] using default parameters (eg. allowing a maximum of 4% alignment mismatch). The individual aligned sam files were converted to their binary form (bam), merged, and sorted using Picard tools 1.69 prior to importing into GATK 3.4 [18] for variant calling. We allowed GATK (Unified Genotyper) to call SNPs with minimal filters, including requiring a base quality score of at least 30 (-stand_call_conf 30.0 -stand_emit_conf 10.0), and a prior on heterozygosity of 0.01 (-hets 0.01). Raw variant call files were then filtered with vcftools 0.1.13b [19] to allow bi-allelic SNPs only, a sequence depth of 8 reads (—minDP 8) for a genotype to be called, a minimum distance between neighbouring SNPs of 10 bp (—thin 10), a maximum of 20% missing data per individual sample and locus (—max-missing 0.80). To remove potential paralogous loci, we discarded SNPs having mean read depths above the 90th percentile of the empirical mean read depth distribution across all loci. SNPs with extreme deviations (p < 0.0001) from Hardy-Weinberg equilibrium (eg. excessive heterozygosity) were also removed. Filtered vcf files were converted to PLINK ped/map format [20] for downstream analysis.

### Identifying clones, polyploids and first degree relatives using GBS

Initially, we performed GBS on 363 accessions which yielded 29,494 SNPs. Next, we restricted our analyses to SNPs with a minor allele frequency (MAF) >0.05, which resulted in 15,802 SNPs. Of these, 14,841 SNPs (93.9%) were mapped to the assembled portion of chromosomes 1–17 of the Golden Delicious genome version 1.0p (www.rosaceae.org). We calculated identity-by-descent (IBD) for all pairs of samples using PLINK. We considered two accessions to be clones of each other when the IBD ($\hat{\pi }$) was >0.85. In theory, IBD = 1 for pairwise clonal relationships. However, two factors can result in IBD < 1 for pairs of accessions that are clonally related. First, reductions in IBD can result from genotyping errors, which likely result primarily from the poor quality of the reference genome: paralogous regions of the genome are collapsed and thus appear as single copy regions in the reference genome so that sequence coverage variation between samples results in different genotype calls between clones. Second, it is possible that somatic mutations between clones exist and these result in a reduction of IBD values. Even with high-quality genotype data from a genotyping microarray, IBD values as low as 0.95 for clonal relationships were previously found in grapes [21]. Considering the uncertainty of the genotype calls with the use of GBS and a relatively poor quality reference genome, we argue that it is reasonable to observe IBD values as low as 0.85 for pairs of accessions that are clonally related. Finally, given the distribution of IBD among all pairwise comparisons, the most parsimonious explanation for the clear bump at the top in the distribution is that these represent clonal relationships.

Next, we used the network package in R to calculate a network adjacency matrix in which pairwise comparisons with ($\hat{\pi }$) >0.85 were indicated with a ‘1’ and all other comparisons were indicated with a ‘0’. We visualized clonal relationships using this matrix and the ‘plot.network’ function in the network package [22]. Before identifying first-degree relationships, we kept only one representative from each clonal group at random. Next, we calculated the observed heterozygosity by individual using the–het function in PLINK and plotted the results, observing a bi-modal distribution which allowed us to easily identify polyploid accessions due to excess heterozosity. Polyploids were excluded from further analysis. Thus, the final data set included 248 unique, diploid genotypes from the original 363 accessions.

After the removal of duplicate clones and polyploids, we repeated the IBD analysis in order to identify first-degree relationships [23, 24]. Accessions with well-known pedigrees such as ‘Aroma’, ‘Discovery’, ‘Elstar’, ‘Gloster’, ‘Ingrid Marie’ and ‘James Grieve’ were used to calibrate the expected range of IBD values for first-degree relationships. Reported first-degree relationships had IBD values ranging from 0.43 to 0.52, and thus, we considered all accessions with pairwise values in this interval to be putative first degree relatives. We used these thresholds to create a network adjacency matrix and visualized the results using the ‘plot.network’ function in the network package in R [22].

In order to examine the population structure, we initially used PLINK to filter for unique, diploid *Malus domectica* accessions which left us with 234 individuals. After filtering for 5% MAF and pruning for LD (command:—indep-pairwise 10 3 0.5), 10459 SNPs remained for analysis. Using fastSTRUCTURE [25] we tested K = 1 to K = 8 and used the "choosek" function to determine the optimal K value, which we selected as K = 1.

### SSR genotyping

SSR genotyping using 15 SSR markers was previously performed on 485 accessions, which included the 363 accessions genotyped using GBS in this study [15]. In the previous work, we identified first-degree relationships using the software CERVUS [26] with a LOD score threshold of 95% [15].

### Examining population structure using PCA

A large number of the studied cultivars derive from few major ancestors, which resulted in distinct genetic clustering shown in previous SSR-based study [15]. Therefore, in the principal components analysis (PCA) we decided to include only two offspring from these major ancestors, ‘Cox Orange, ‘Pigeon blanc’ and ‘Melonenapfel’. In addition, for the SSR data, accessions with >20% missing data across the 15 SSRs examined were removed from the dataset. Population structure among the remaining 204 accessions was investigated using the adegenet package [27, 28] in R v.3.3.2 [29]. The ‘scaleGen’ function was used to replace missing data by the mean allele frequencies. PCA was performed using the ‘dudi.pca’ function, while centering and scaling the data, and accessions were labelled according to species. Subsequently, accessions labelled as *Malus sieversii* were removed from the data set and accessions with >20% missing data were removed, resulting in 190 *M*. *domestica* accessions. Missing data was replaced by mean allele frequencies and PCA was performed again. *M*. *domestica* accessions were labelled according to origin and harvest time.

PCA was also performed using the SNP genotypes. First, the 204 accessions included in the SSR analysis were extracted from the genotype table using PLINK [23, 24]. Missing data was imputed using LinkImpute (parameters: *k* = 3, *l* = 18) and the resulting imputation accuracy was 93.7% [30]. SNPs with a minor allele frequency (MAF) <0.01 were removed, reducing the SNP set from 24,533 SNPs to 23,446. SNPs were then pruned for linkage disequilibrium using PLINK (—indep-pairwise 10 3 0.5) [23, 24], reducing the number of markers from 23,460 to 17,737 for PCA. The resulting SNP genotype data was analyzed using the same method as the SSRs: by centering and scaling the data using the ‘dudi.pca’ function in the adegenet package [27, 28] in R v.3.3.2 [29]. We divided accessions based on species and used a Mann-Whitney U test to estimate if species differed along the SSR and SNP PC1 and PC2.

Next, we extracted the 190 *M*. *domestica* accessions from the imputed SNP dataset and repeated the MAF filter of 0.01 and LD-pruning using PLINK [23, 24]. The number of SNPs was reduced from 24,533 SNPs to 17,700, after which PCA analysis was repeated with accessions labelled according to origin and harvest time. We divided accessions based on origin and used a Mann-Whitney U test to estimate if accessions differed along SNP PC2 based on origin. Finally, we tested the correlation between SSR PCs 1 to 5 and SNP PCs 1 to 5, as calculated using all 204 accessions. We used a Pearson’s correlation and all p-values were Bonferroni-corrected (multiplied by 25) for multiple comparisons. All PCA results were visualized using the ggplot2 package in R [31].

## Results

GBS yielded on average 2.5 million sequence reads per sample for the 363 accessions, with a coefficient of variation of 17%. The accessions genotyped using GBS reflected a subset of accessions previously genotyped using SSR markers. Ploidy levels, determined using flow cytometry, were also available for accessions included in the previous work [15]. This enabled us to compare the strength of GBS, SSRs, and flow cytometry for identifying clones, ploidy levels, establishing first-degree relationships and revealing the underlying genetic structure of the accessions.

### GBS reveals triploid accessions

**Fig 1:**
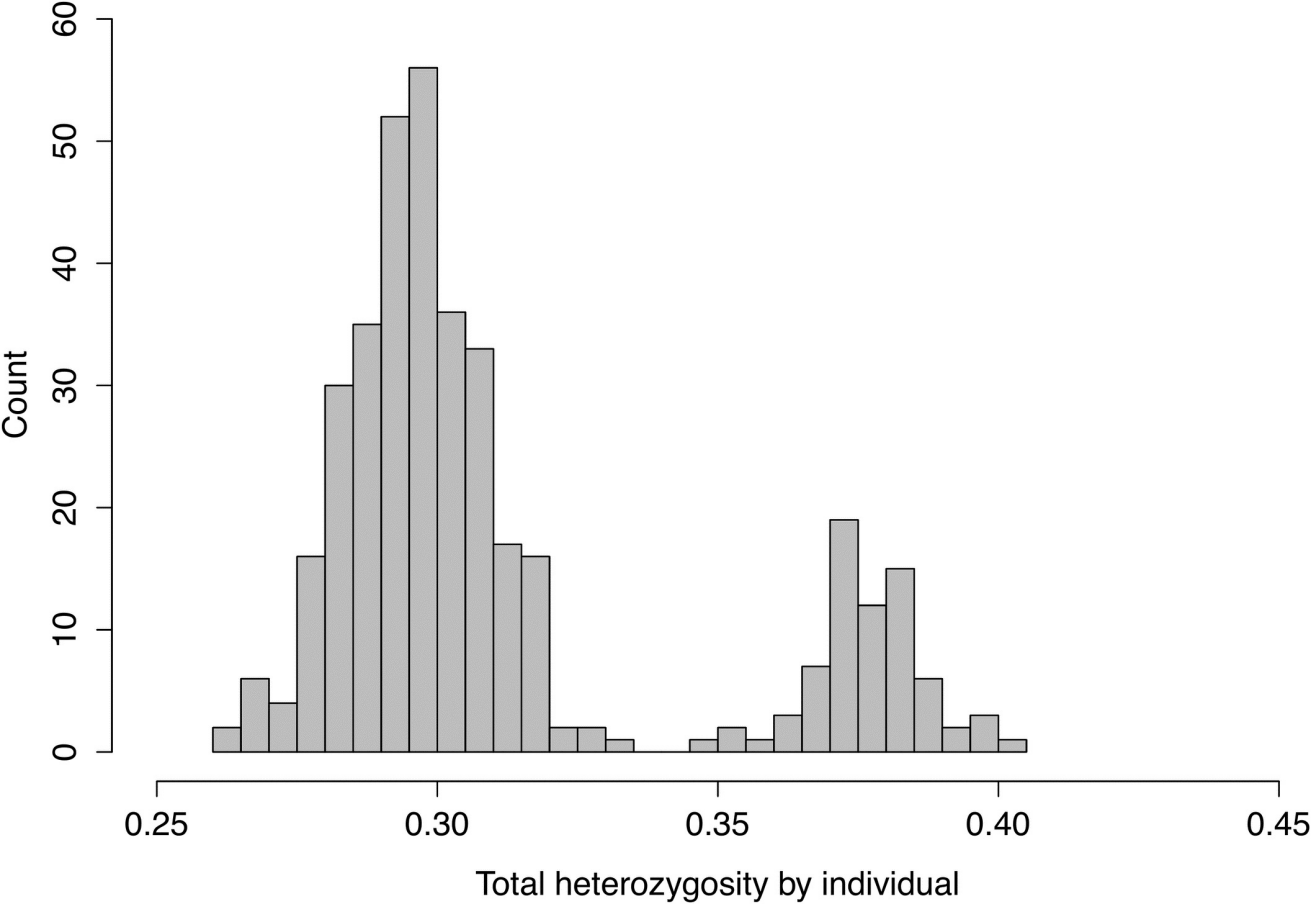
Bar plot of heterozygosity by individual among 15,802 SNPs generated by GBS. The first cluster (heterozygosity ≤ 0.335) contains all diploid accessions whereas the other cluster (heterozygosity ≥ 0.345) comprises triploid accessions according flow cytometry analysis.

Using genome-wide SNP data, we calculated total heterozygosity by individual, which separated accessions into two groups with heterozygosity ≤ 0.335 or ≥ 0.345 (Fig 1). We compared the accessions in each of the two groups with ploidy levels previously established by flow cytometry [15] and found that accessions with heterozygosity ≤ 0.335 were diploid according to flow cytometry data and that accessions with heterozygosity ≥ 0.345 were triploid. Ploidy levels revealed by both GBS and flow cytometry are given in S1 Table.

### Relationships and population structure

**Fig 2:**
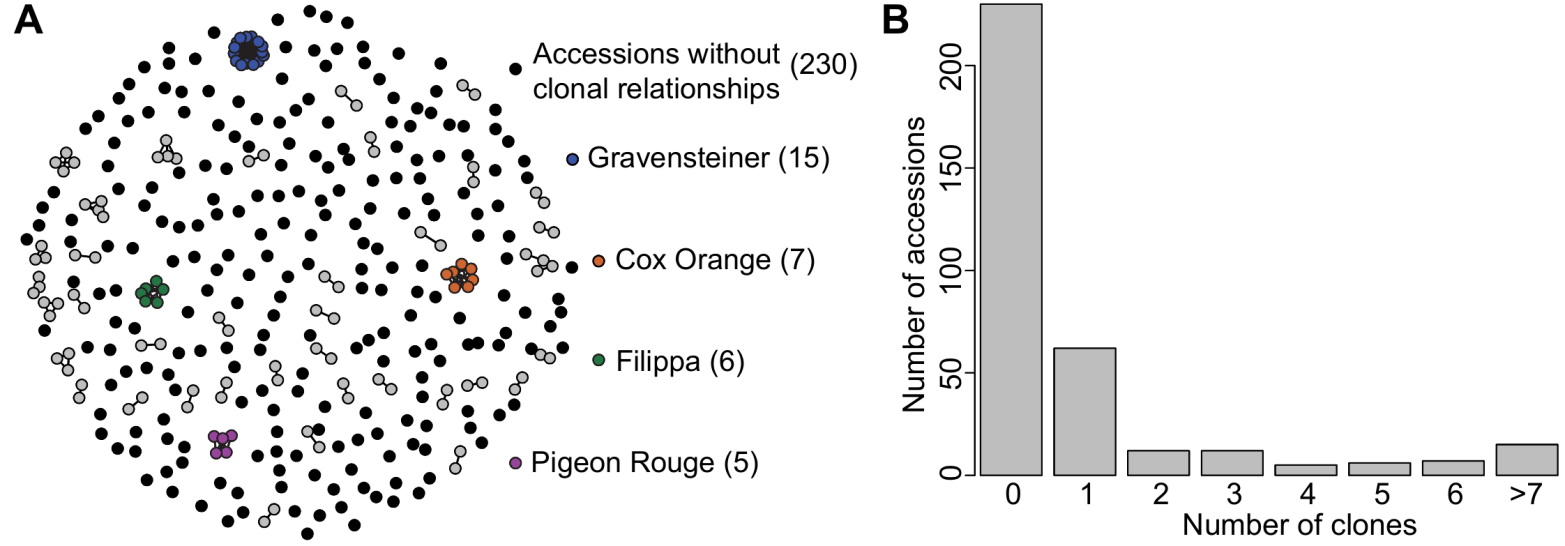
Clonal relationships in the Danish apple germplasm collection. (A) Network of clonal relationships among 349 *Malus domestica* accessions. Each accession is represented by a dot. Accessions without a clonal relationship to other accessions are indicated with a black dot. Accessions with four or fewer clonal relationships are shown in grey together with their clones. Accessions with five or more clonal relationships are indicated by a colour code. (B) For each of the 349 accessions, the number of clonal relationships was evaluated. The majority of accessions (230) are without clonal relationships while 119 (34%) of the accessions have clonal relationships with one or more accessions.

We found 230 accessions without any clonal relationships and 119 accessions with at least one putative clonal relationship, resulting in a total of 272 unique genotypes. For some cultivars, somatic mutations have resulted in several clones, such as colour sports, that have been maintained through grafting. We identified 42 putative clonal groups, of which the majority (31) consists of two clonal accessions. The highest number of accessions within a clonal group was 15, which were identified for ‘Gravensteiner’ (Fig 2).

**Fig 3:**
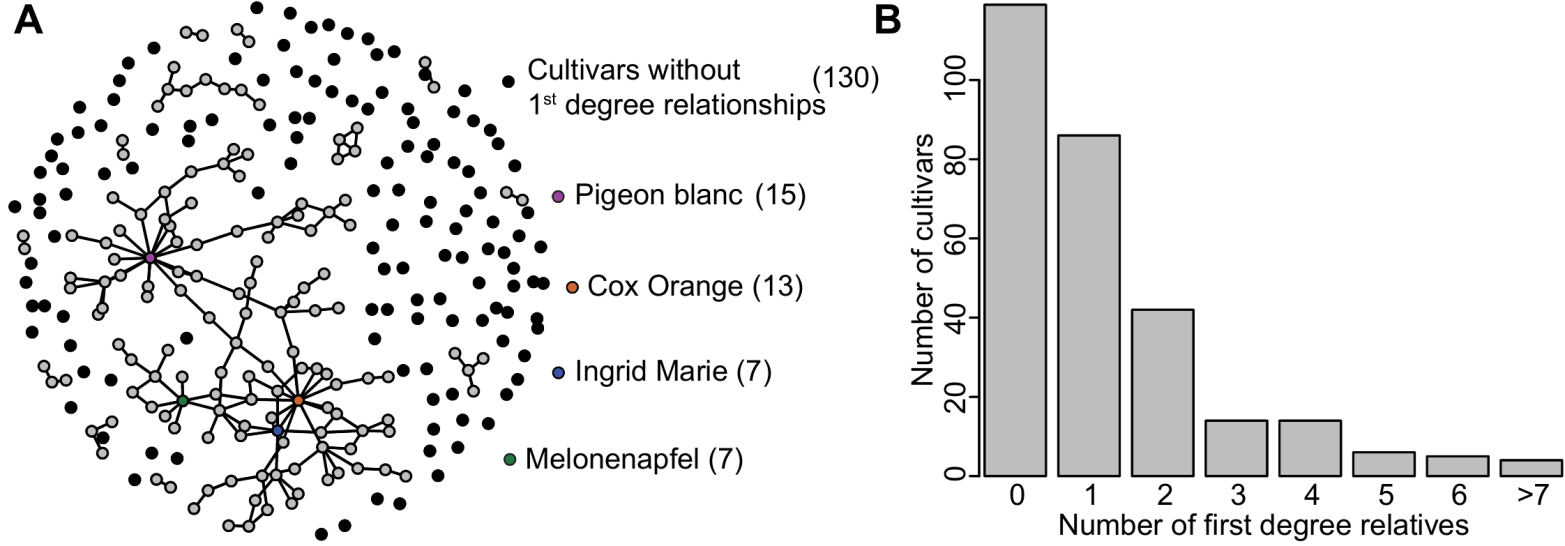
Pedigree structure in the Danish apple germplasm collection. (A) Network of first-degree relationships for each of the 272 unique *Malus domestica* cultivars. Each unique apple cultivar is represented by a dot and edges in the network represent first-degree relationships. Cultivars without first-degree relationships in the collection are indicated by a lone, black dot. In total, 142 cultivars have at least one first-degree relationship. The largest interconnected network includes 106 (39%) of the unique cultivars that are connected through a series of first-degree relatives. (B) The number of first-degree relationships for each of the 272 cultivars. While 130 (48%) cultivars are without first-degree relationships, 52% of the cultivars have a first-degree relationship with at least one other accession.

Analysis of first-degree relationships revealed 142 (52%) accessions with at least one first-degree relative in the collection (Fig 3 and S2 Table). 106 (30%) accessions form a single network that is inter-connected through a series of first-degree relationships. Of the 154 first-degree relationships identified, the majority (96) were discovered using both SSR and SNP markers. 31 first-degree relationships were identified using SSR markers but not SNP markers, whereas 27 were revealed by SNP markers and not by SSR markers (S2 Table).

**Fig 4:**
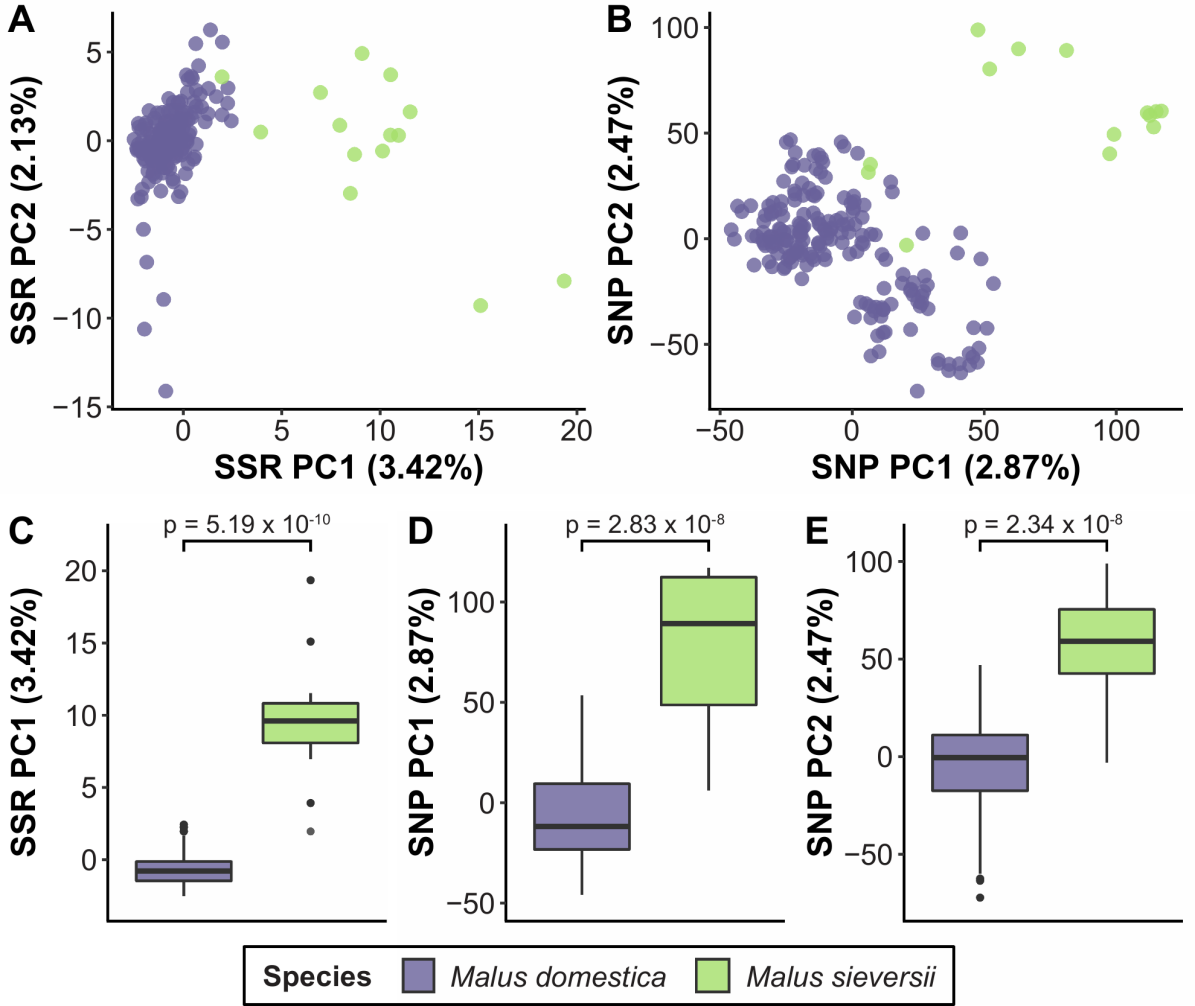
Principal components analysis (PCA) based on (A) SSR and (B) SNP data for 204 accessions. Accessions are labelled based on species, and boxplots of species distribution along (C) SSR PC1, (D) SNP PC1 and (E) SNP PC2 are included. The percentage of variance explained by each PC is indicated in parentheses. Results from Mann-Whitney U-tests between *Malus domestica* and *M*. *sieversii* are also reported.

Differentiation between *Malus domestica* and *M*. *sieversii* was found for SSR-based analysis along PC1 (p = 5.19 × 10^−10^); and based on SNP data along PC1 (p = 2.83 × 10^−8^) and PC2 (p = 2.34 × 10^−8^) (Fig 4). The genomic PC positional information for all accessions are listed in S3 Table. Labelling *M*. *domestica* accessions according their harvest time resulted in no significant separation; whereas accessions of Danish origin vs. other geographical origins differed using SNP-based PCA along PC2 (p = 0.002) (S1 Fig).

## Discussion

Next generation sequencing combines high-throughput SNP-discovery and genotyping, resulting in high-density SNP-marker data. It is currently replacing traditional genotyping techniques, like SSR-markers, primarily because of its ease and its suitability for GWAS and genomic selection. Genome re-sequencing provides higher resolution, but for many purposes fewer markers are sufficient. Thus, various approaches have been developed to reduce sequencing costs, either by focusing on expressed sequences through RNAseq or on sequences next to restriction enzyme sites. There are several variants of the former approach including GBS [7], RAD-seq [32], SBG [33], and DArTseq [34].

Here we genotyped 349 apple accessions using the GBS protocol [7] and compared the resulting information with our previous investigations using SSR markers and flow cytometry [15]. When we filtered for less than 20% missing data per individual sample and locus, and a MAF > 5%, 15,802 SNPs remained and 14,841 SNPs (93.9%) of these were mapped to chromosomes 1–17 of the Golden Delicious genome version 1.0p. This is considerably more than the 8,657 SNP-markers obtained with the same GBS-protocol in the USDA apple germplasm collection [8] and also much more than the ~4000 SNPs found among F1 progenies using RADseq [35] or GBS [16]. The reason for this high number of SNPs is unlikely to be that the Danish apple collection contains higher levels of genetic diversity. A more likely explanation lies in technical improvements. We carefully equilibrated DNA concentrations before multiplexing which likely resulted in more even coverage across samples and hence fewer SNPs removed due to missing data. Future improvements to genomic resources (e.g. the apple reference genome assembly and genotype imputation algorithms [30, 36]) will allow for higher numbers of SNPs, as evidenced by recent work in apple which identified 122,000 SNPs using GBS [37].

### Using GBS to identify ploidy levels

Triploid apples are relatively common among cultivars: they have a large fruit size and vigorous growth. We identified 19% of the accessions examined as triploids, which are assumed to be the result of fusions between unreduced diploid gametes and normal haploid gametes. Triploid individuals generally have a 50% higher level of heterozygosity than diploid individuals, assuming Hardy-Weinberg equilibrium conditions, independently of the allele frequency. We found a clear difference in the level of heterozygosity between diploid and triploid accessions (Fig 1). However, it was only about 30% higher in triploids, likely because the studied collection is not a population in Hardy-Weinberg equilibrium. Determination of ploidy level in aspen (*Populus tremuloides* Michx.) was recently described [38] by applying a relatively complex statistical model to GBS data. However, here we show that triploids can be differentiated from diploids simply by the level of heterozygosity in a diverse collection of apple. In other apple germplasm, where triploids are derived from a limited set of diploids, ploidy levels may be indistinguishable. Our use of heterozygosity to distinguish ploidy levels therefore warrants further investigation in other collections of apples and other species. The Infinium SNP-array platform can also be used to determine ploidy level and aneuploidy in apple due to variations in the ratio of signal intensity from the two alleles of each SNP along the chromosomes [39]. However, we do not have information concerning aneuploidy in our collection because it is not easily revealed by flow cytometry.

### SSRs vs. SNP-markers for revealing parentages

The number of SSRs and SNP-markers needed to achieve a sufficiently high probability of correct identification of first-degree relationships depends on the level of heterozygosity and the number and frequency of alleles. However, based on theoretical estimations [40, 41] about 5–10 SNPs equals one SSR and about 200 SNP markers are needed to characterize relatedness. The high number of alleles for SSR-markers makes each individual SSR-marker much more informative than bi-allelic SNP genotypes. Identification of clones using SSR markers was performed in a previous study [15] and we found the exact same clonal relationships using SSR markers and SNP markers (S1 Table). Hence, we found the two approaches equally effective for identification of clones. Identifying pedigree relationships, however, is more complicated and we therefore find several reasons to compare the strength of 15 SSR-markers with more than 15,000 SNPs.

Firstly, the use of SSRs is still the gold standard due to the large number of alleles, the high reliability of the resulting genotype calls, the transferability between studies, and the relatively small number of markers required to unambiguously reveal pedigree relatedness. The use of GBS markers for pedigree analysis is, however, still in its infancy and, due to the genotype uncertainty and the relatively low pedigree-relevant informativeness per SNP, there is still uncertainty about where to draw thresholds for pedigree relatedness and what metrics of relatedness (IBD or IBS or other) to use. In addition, pedigree relatedness in a highly heterozygous and clonally propagated organism like apple is severely complicated due to the nature of the pedigree relations: an individual can cross with its own grandparent, cousin etc. and popular cultivars end up being extensively crossed such that their alleles become highly overrepresented in the population overall. This was observed here, were ‘Cox’s Orange’ is a frequent parent to many of the studied cultivars.

Nevertheless, from manually checking the SSR markers and from historical reports on parentages, we conclude that first-degree relationships revealed by both marker systems are parent-offspring relations. Relationships revealed by only one of the two marker systems are primarily first-degree relations with examples of second degree relations. The second degree relations are dominated by half siblings that have ‘Cox’s Orange’ as one parent. The studied accessions includes the most important cultivars that have been grown in Denmark during the past centuries [42–44], such as ‘Pigeon blanc’, ‘Cox’s Orange’ and ‘Ingrid Marie’ and these diploid cultivars also have the highest number of first-degree relationships (Fig 3). Our findings are therefore consistent with historical information where the reported place and year of origin [42, 44, 45] has helped us pinpoint the putative parent and offspring in many first-degree relations. We found that ‘Melonenapfel’, described for the first time in 1788 [46], is the parent of the two important Danish cultivars, ‘Filippa’ and ‘Dronning Louise’ (S2 Table).

### Gene bank population structure

We found differentiation between accessions of Danish origin and other geographical origins on the basis of the SNP data (S1 Fig). This was not observed for the SSR data. The primary reason for this discrepancy between the SSR and SNP data is likely marker number. The 15 SSR markers provide a far less comprehensive view of the entire genome than the 15k SNPs. With 17 chromosomes, 15 SSRs do not even provide a marker for every chromosome, and given the rapid LD decay and short haplotype blocks observed in diverse collections of apples [8], 15 markers is expected to provide a snapshot of roughly <0.01% of the segregating haplotypes in the population. Thus, the SNP data are a far more powerful system to detect structure, as described from other studies e.g. [47, 48], and were able to detect even the relatively weak differentiation we observe between Danish and other apple cultivars (S1 Fig).

No well-defined subpopulations were identified within the *Malus domestica* accessions of Danish origin, which is in line with previous findings based on SSR data [15]. Also fastSTRUCTURE analysis suggests that K = 1 gives the best description of population structure and thereby support previous findings [15] that population structure is lacking in the *M*. *domestica* collection. At the species level, *M*. *domestica* and *M*. *sieversii* differentiate, which is in accordance with previous findings [8] from the USDA-Germplasm collection. In contrast to this previous work, we did not find that population structure was correlated with harvest time (S1 Fig). This is probably because the accessions studied here do not represent as broad a sample as the USDA collection but rather represent a genetic group of apples adapted to North European costal climate with a short harvest window.

### Comparison SSR and SNP marker approaches

An accurate cost comparison between SSR-based marker analysis and GBS-based SNP-marker analysis is difficult because many laboratory-specific conditions will influence the cost, such as the price of labour and availability of instruments. In our study, sequencing costs for the genome-wide SNP data were at least 10 times more expensive than the laboratory costs of obtaining the SSR data. However, obtaining the final SSR genotype data is much more labour-intensive since it requires preparing a large number of PCR reactions, performing allele calling, and many more additional (manual) steps.

Prediction of ploidy levels based on the degree of heterozygosity of the SNPs, as observed here, may not account for all collections of apple and thus, needs confirmation by flow cytometry. However, it may well be the case that the degree of heterozygosity clusters in two clearly separated groups. We therefore hypothesize that the use of genome-wide SNP data using next-generation sequencing will be more desirable and efficient for future characterization of germplasm collections. Finally, even though both SSR- and SNP-markers are powerful tools for exploring genetic diversity, only GBS or high-density SNP-arrays provide enough SNP-markers for GWAS, which can then enable marker-assisted breeding. Therefore, despite uncovering similar results in our work using both SSRs and SNPs, most future work will benefit from using SNPs, which allow for both GWAS and detailed population studies to be performed.

## Supporting information

S1 Table Accession list including clonal groups and ploidy levels. (XLSX)

S2 Table First-degree relations revealed by SSR and SNP markers. (XLSX)

S3 Table Positional information for SSR and SNP PCs visualized in Fig 4. (XLSX)

S1 Fig PCA analysis for *Malus domestica* accessions. PCA plot made on basis of SSR-based analysis (A) and SNP-based analysis (B) which enabled to distinguish between accessions of Danish origin and other geographical origins (C).
(PDF)

S2 Fig Comparisons of SSR and SNPs using PCs with Bonferroni correction. (PDF)
