# Supplementary figures and images for: Population structure, relatedness and ploidy levels in an apple gene bank revealed through genotyping-by-sequencing
Source: PLoS One. 2018 Aug 15;13(8):e0201889. doi: 10.1371/journal.pone.0201889 (PMC6093671; doi:10.1371/journal.pone.0201889)

**A**

SSR PC2 (2.37%)

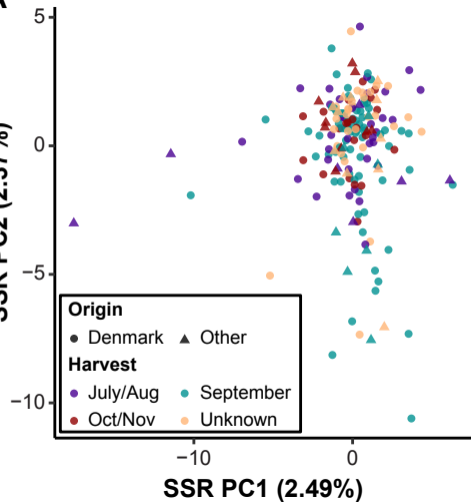**B**

SNP PC2 (1.91%)

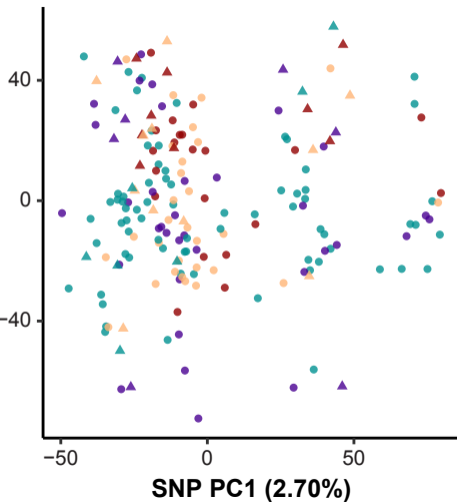**C**

SNP PC2 (1.91%)

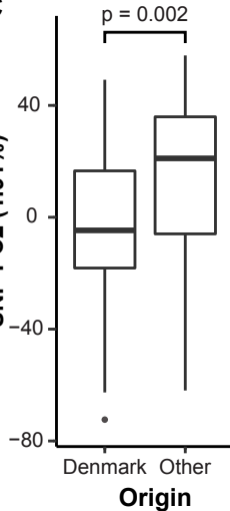

Supplement: S1 Fig — PCA plot made on basis of SSR-based analysis (A) and SNP-based analysis (B) which enabled to distinguish between accessions of Danish origin and other geographical origins (C). (PDF) [file pone.0201889.s004.pdf]

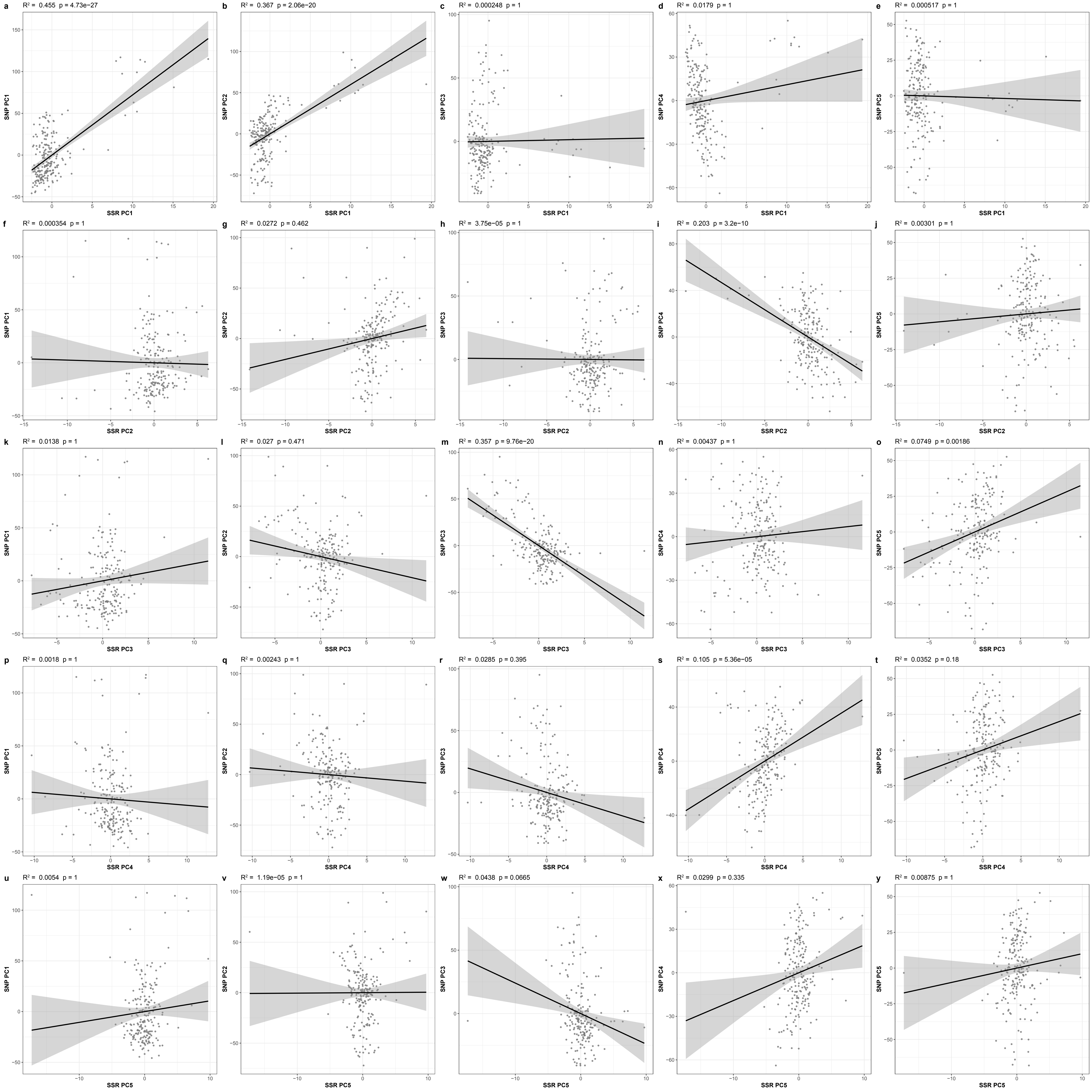

Supplement: S2 Fig — (PDF) [file pone.0201889.s005.pdf]
